# Mitochondrial deficits in human iPSC-derived neurons from patients with 22q11.2 deletion syndrome and schizophrenia

**DOI:** 10.1038/s41398-019-0643-y

**Published:** 2019-11-18

**Authors:** Jianping Li, Sean K. Ryan, Erik Deboer, Kieona Cook, Shane Fitzgerald, Herbert M. Lachman, Douglas C. Wallace, Ethan M. Goldberg, Stewart A. Anderson

**Affiliations:** 1 Department of Psychiatry, Children’s Hospital of Philadelphia, Philadelphia, PA USA; 2 Department of Psychiatry, The Children’s Hospital of Philadelphia and the University of Pennsylvania Perelman School of Medicine, Philadelphia, PA USA; 3 Mallinckrodt Pharmaceuticals, Bedminster, NJ USA; 4 University of Pennsylvania, Philadelphia, PA USA; 5 Department of Psychiatry, Children’s Hospital of Philadelphia, Philadelphia, PA USA; 6 Department of Psychiatry and Behavioral Sciences, Albert Einstein College of Medicine, Bronx, New York USA; 7 Center for Mitochondrial and Epigenomic Medicine, Children’s Hospital of Philadelphia and Department of Pediatrics, Division of Human Genetics, Perelman School of Medicine, University of Pennsylvania, Philadelphia, PA United States; 8 Department of Pediatrics, The Children’s Hospital of Philadelphia and the University of Pennsylvania Perelman School of Medicine, Philadelphia, PA USA; 9 Department of Psychiatry, Children’s Hospital of Philadelphia and the University of Pennsylvania Perelman School of Medicine, Philadelphia, PA USA

**Keywords:** Molecular neuroscience, Schizophrenia

## Abstract

Schizophrenia (SZ) is a highly heterogeneous disorder in both its symptoms and risk factors. One of the most prevalent genetic risk factors for SZ is the hemizygous microdeletion at chromosome 22q11.2 (22q11DS) that confers a 25-fold increased risk. Six of the genes directly disrupted in 22qDS encode for mitochondrial-localizing proteins. Here, we test the hypothesis that stem cell-derived neurons from subjects with the 22q11DS and SZ have mitochondrial deficits relative to typically developing controls. Human iPSCs from four lines of affected subjects and five lines of controls were differentiated into forebrain-like excitatory neurons. In the patient group, we find significant reductions of ATP levels that appear to be secondary to reduced activity in oxidative phosphorylation complexes I and IV. Protein products of mitochondrial-encoded genes are also reduced. As one of the genes deleted in the 22q11.2 region is *MRPL40*, a component of the mitochondrial ribosome, we generated a heterozygous mutation of *MRPL40* in a healthy control iPSC line. Relative to its isogenic control, this line shows similar deficits in mitochondrial DNA-encoded proteins, ATP level, and complex I and IV activity. These results suggest that in the 22q11DS *MRPL40* heterozygosity leads to reduced mitochondria ATP production secondary to altered mitochondrial protein levels. Such defects could have profound effects on neuronal function in vivo.

## Introduction

Schizophrenia (SZ) is a heterogeneous disorder generally characterized by adolescent onset of psychosis together with deteriorations of social and cognitive functioning^1,2^. Metabolic compromise, including mitochondrial dysfunction, has long been posited to contribute to the development of symptoms in SZ^3,4^. Recent studies have further strengthened this notion. For example, gene expression alterations in postmortem neocortical pyramidal neurons suggest disruption of mitochondrial function^5^, and other studies reported mitochondrial defects in neural progenitors and cortical interneurons (cINs)^6^ derived from induced pluripotent stem cells (iPSCs) from patients with SZ relative to healthy controls^7^. However, despite the potential importance to the discovery of novel treatments for SZ, evidence for mitochondrial dysfunction in SZ in living, human neurons with a defined genetic alteration has been lacking.

The most prevalent genetic risk factor for SZ is the 22q11.2 deletion syndrome (22q11DS), occurring in about 1:4000 births^8^. Roughly one-quarter of people with the 22q11DS, a 25-fold increase from the general population, develop SZ in a manner that is not grossly distinguishable from non-syndromic SZ^9,10^. Remarkably, in 22q11DS, six of the ~45 deleted genes encode for proteins that are mitochondrial-localizing, and three others strongly influence mitochondrial function^11,12^. One study has found evidence for mitochondrial dysfunction in serum samples from subjects with the 22q11DS^12^, while another recent study used proteomic analyses in fibroblasts from 22q11DS subjects, together with analyses of 22q model transgenic mice and flies to conclude that mitochondrial SLC25A1/A4 and TXNRD2 influence synaptic function^13^. The importance of Txnrd2 in long-range cortical connectivity and psychosis-related cognitive deficits was further established recently in transgenic mice^14^. Finally, *MRPL40* was identified as a candidate SZ risk gene^15^, and transgenic mice lacking one copy of Mrpl40 show alterations in mitochondrial calcium as well as psychosis-related cognitive deficits^16^. However, to our knowledge, no evidence for mitochondrial dysfunction in living, human neurons from 22q11DS with SZ has been reported.

Here, we have studied mitochondrial function in iPSC-derived neurons from patients with 22q11DS + SZ (22qSZ) versus healthy controls. Patient-derived neurons have reduced ATP levels, and reduced activity of complexes I and IV of the electron transport chain (ETC). The levels of multiple mitochondrial-translated proteins are reduced, in contrast to the levels of several nuclear-encoded mitochondrial proteins. These findings were replicated in an iPSC line that we edited to be heterozygous for *MRPL40*. These results suggest that defects in mitochondrial ATP production secondary to reduced levels of mitochondrial-encoded proteins may contribute to neuronal dysfunction in the 22qSZ.

## Materials and methods

### Human iPSCs

Human iPSCs were contributed by Herbert Lachman (Albert Einstein University), and all of those from patients with 22q11DS also were diagnosed with SZ^17^. All cell lines had SALSA MLPA KIT P250-A1 DiGeorge (MRC Holland, Amsterdam, Netherlands) testing for the 22q11.2 deletion^18^, and the 22q11.2 deletion lines were confirmed to be hemizygous for the 3 mb deletion located in “A-D” region. In addition, each line was tested monthly and confirmed to be free of mycoplasma. Human iPSCs from four lines of affected subjects and five lines of controls were applied in this study.

### Lentiviral vector generation

Plasmids of VSVG.HIV.SIN.cPPT.CMV.mNgn2.WPRE and VSVG.HIV.SIN. cPPT.CMV.rTTA. WPRE were kindly provided by Dr. Marius Wernig (Stanford University), and packaged into virus by the University of Pennsylvania Viral Vector Core (VSVG.HIV.SIN.cPPT. CMV.mNgn2. WPRE and VSVG.HIV.SIN. cPPT.CMV. rTTA.WPRE.)

### Neuronal differentiation of iPSCs

Induced differentiation of human iPSC lines to neurons (iNrns) was accomplished by an established protocol^19^. In brief, iPSC lines were infected with two lentiviral vectors: TetO-mNgn2-T2A-PuroR and Ubiq-rTTA. On day 1, differentiation was initiated with exposure to doxycycline (2 µg/ml, Sigma), followed by puromycin (5 µg/ml, Sigma) selection for cells that possessed these two lentiviral vectors 24 h later. The cells were replated the next day and grown in Neurobasal-A medium (Gibco) with B27 (Life Technologies), glutamax (Life technologies), 5 mM glucose, 10 mM sodium pyruvate, 10 ng/ml NT-3 (Peprotech), 10 ng/ml Brain-derived neurotrophic factor (Peprotech), and 2 µg/ml doxycycline. Rat glia, if used, were added on day 5, and a single administration of 2 µM Ara-C (Sigma) was added on day 7. Doxycycline was discontinued on day 10. iNrns were cultured until at least day 21.

### Rat glia

Isolated E17 Sprague Dawley rat cortex was obtained from the UPenn NRU Core. Cells were plated onto uncoated 10 cm dishes at 1.5 million cells/ml. Cells were grown in NM-15 media (Eagle’s MEM with Earle’s salts and 2 mM l-glutamine, 15% heat-inactivated fetal bovine serum, 6 mg/ml glucose, 0.5 U/ml penicillin, and 0.5 μg/ml streptomycin). Medium exchanges occurred every 5 days, and rat glia grew for at least 2 weeks. Prior to glial harvest, the glia were shaken at 37 °C for 3 h at 230 rpm to remove microglia. The glia were then dissociated with accutase (Life Technologies), spun down for 5 min at 1000 rpm, resuspended in neurobasal/B27 media, and plated onto the neurons at 100k cells/well of a 24-well plate.

### Western blot

Cells were washed with ice-cold PBS twice. Ice-cold RIPA buffer and protease inhibitor cocktail were added next, and cells were scraped from the plate and put into a 1.5-ml Eppendorf tube. Cells were placed on ice for 30 min, then centrifuged at 14,000 rpm for 15 min at 4 °C. Supernatant was collected and frozen at −80 °C until needed. Protein concentration was quantified with the Pierce BCA kit and a NanoDrop2000 (Thermo scientific) to measure. Samples were loaded on a 4–12% bis-tris gel with LDS Sample Buffer (Life technologies) and Sample Reducing Agent (Life technologies). The SDS-PAGE was run with MOPS buffer (Life Technologies), protein was transferred from gel to nitrocellulose membrane, which was then blocked for 1 h at room temperature using 5% BSA. Li-Cor/Odyssey and Image J are used for data collection and analysis. We used the following primary antibodies: anti-MRPL40 (1:500; Novus), anti-VDAC (1:1000; Neuromab), anti-cytochrome b (1:200; Santa Cruz Biotechnology), anti-MT-ND1 (1:500; Abcam), anti-OXPHOS cocktail (1:250; abcam), and β-actin (1:10,000; Cell Signaling Technology). The following Licor secondary antibodies were used all at 1:10,000: IRDye 680LT Goat anti-Mouse, IRDye 680RD Donkey anti-Rabbit, IRDye 800CW Donkey anti-Rabbit, IRDye 800CW Donkey anti-Goat, IRDye 800CW Donkey anti-Mouse.

### RNA extraction, reverse transcription, and quantitative PCR

Total RNA was extracted using Trizol (Ambion) and the concentration was measured using nanoDrop2000 spectrophotometer. cDNA was generated using transcript IV VILO Master Mix (Thermo Fisher). RNA abundance was measured by Quantitative PCR using TaqMan Gene Expression Master Mix (Applied Biosystems) for primer pairs of ACTIN, MRPL40, VDAC1, and COX1 purchased from Thermo Fisher Scientific, and SYBR Green PCR Mix (Applied Biosystems) for primer pairs of cytochrome b and mt-ND1. Primer sequences: ACTIN (Assay ID: Hs01060665 _g1), MRPL40 (Assay ID: Hs00186843_m1), VDAC1 (Assay ID: Hs01019083 _m1), COX1 (Assay ID: Hs02596864_g1); cytochrome b: forward primer: 5′-AGTCCCACCCTCACACGATTCTTT-3′, reverse primer: 5′-AGTAAGCCGAGGGCGTCTTTGATT-3′; mt-ND1: forward primer: 5′-ATGGCCAACCTCCTACTCCTCATT-3′, reverse primer: 5′-TTATGGCGTCAGCGAAGGGTTG TA-3′.

### Synapse counting

Induced neurons grown to day 21 with rat glia were fixed in 4% PFA, stained with DAPI (1:2000; Invitrogen), VGLUT1 (1:1000; Sigma), PSD95 (1:500; Neuromab), and MAP2 (1:500; Abcam). The respective secondary antibodies were Donkey Anti-Rabbit Alexa 488 (1:500; Thermo Fisher Scientific), Goat anti-Mouse Alexa 568 (1:500; Thermofisher Scientific), and Goat anti-Chicken 680 (1:500; Thermo Fisher Scientific). Then, 40× images were collected using a Leica DMI8 confocal microscope. At least three images were collected from three control and three schizophrenic lines of cells. Each neuron imaged had no less than two major processes. After collection, images were deconvolved using Hyguens Essential software. Next, images were transferred to Imaris software, where they were then cropped and surfaced by MAP2 staining. PSD95 puncta about 0.5 µm in diameter were identified within the MAP2-positive dendrites, then VGLUT1 puncta of 0.5 µm localized within 1 µm of the PSD-95 were identified. These co-localized puncta within 40 µm of the cell soma were quantified as synapses. Cells were counted to normalize for culture density by selecting random areas and counting number of DAPI+MAP2+ cells per unit area.

### Preparation of cell lysate for mitochondrial OXPHOS activity

The preparation of cell lysate was conducted as published^20^. Briefly, induced neurons at day 21 were washed with cold PBS, then suspended and centrifuged at 1000 rpm for 5 min. Cell pellets were stored at −80 °C until measurement of OXPHOS activity.

### Measurement of mitochondria OXPHOS activity and ATP levels

Biochemical methods were used as described^21^. Briefly, cell pellets were thawed, then flash frozen in liquid nitrogen three times. Cell lysates were placed into a cuvette with reaction buffer and recorded at the relevant wavelength for each complex assay. Potassium buffer was used in all enzyme activity assays and contains 50 mM KCl, 10 mM Tris-HCl, and 1 mM EDTA with pH 7.4. Reagents for detection of complex I enzyme activity (wavelength 340 nm) include 5 mM MgCl_2_, 2 mM KCN, 0.13 mM fresh NADH, 65 µM CoQ_1_, 2 µg/ml antimycin, and 2 µg/ml rotenone. Reagents for complex II activity (wavelength 600 nm) consist of 5 mM MgCl_2_, 20 mM succinate, 2 mM KCN, 65 µM CoQ_2_, 2 µg/ml antimycin, 2 µg/ml rotenone, and 50 µM dichlorophenolindophenol. Reagents used for complex III activity (wavelength 550 nm) include 5 mM MgCl_2_, 2 mM KCN, 15 mM fresh cytochrome c, 65 µM CoQ_2_, 0.6 mM dodecyl-β-d-maltoside, and 2 µg/ml rotenone. The reagent used for complex IV activity (wavelength 550 nm) is 15 µM cytochrome c. Complex V (ATP synthase) enzyme activity Microplate Assay Kit (Abcam, ab109714) was applied following manufacturer’s instructions. All chemicals for enzyme analyses were purchased from Sigma-Aldrich.

ATP levels were quantified on a Luminescence plate reader using an ATP Bioluminescence Assay Kit (Abcam) following the manufacturer’s instructions. Briefly, cells were lysed by detergent provided in the kit, followed by addition of reconstituted substrate solution and measurement of luminescence. All procedures were performed in the dark.

### Generation of *MRPL40* heterozygous line

The *MRPL40* heterozygous line was generated as described^22^. Guide RNAs were designed using http://crispr.mit.edu to locate to exon 2 of *MRPL40*. Guide RNAs were ligated into Fast BbsI digested pSpCas9 (BB)-GFP vectors (Addgene, plasmid ID: 48138). The cloned gRNA–Cas9n vectors were introduced into human iPSCs by electroporation under program B16. Human stem cell Nucleofector Kit1 (Lonza) was used. SURVEYOR assays (Transgenomic, cat. no. 706025), DNA sequencing, and western blot were used to validate the MRPL40 heterozygous mutation. Sequence of gRNAs: gRNA-Top: 5′-CACCGAAGACAACAATGACGCTCGC-3′; gRNA-Bottom: 5’AAACGCGAGC GTC ATTGTTGTCTTC-3′. MRPL40 primers: Forward primer: 5′-CCTTCCACGTTGACCTTGCT-3′; Reverse primer: 5′-CCTTCCACGTTGACCTTGCT-3′.

### Mitochondrial DNA copy number

All reactions were performed in fast optical 96-well reaction plates with barcodes (Applied Biosystems) on SDS7900HT system (CHOP NAPCore). Each sample was analyzed in triplicate. The reaction solution contains 2 μl of DNA template (3 ng/µl), 2 µl of primers (5 µm), 12.5 µl of SYBR Green PCR Master Mix (Applied biosystems), and 8.5 µl of H_2_O. The procedure of amplification program was as follows: 10 min at 95 °C, 40 cycles of 15 s at 95 °C, and 60 s at 60 °C and melting curve. Relative mtDNA copy number (mtDNA amount/nDNA amount) was calculated by a comparative Ct method, using the following equation: mtDNA/nDNA = 2^−ΔCt^. Nuclear primers includes LPL-F: 5′-CGAGTCGTCTTTCTCCTGATGAT-3′, LPL-R: 5′-TTCTGGATTCCAATGCTTCGA-3′, B2-microglobulin-F: 5′-TGCTGTCTCCATGTTTGATGTATCT-3′ and B2-microglobulin-R: 5′-TCTCT GCTCCCCACCTCTAAGT-3′. Mitochondria primers contain tRNA Leu(UUR)-F: 5′-CACCCAAGAACAGGGTTTGT-3′, tRNA Leu(UUR)-R: 5′-TGGCCATGGGTATGTTGTTA-3′; ND1-F: 5′-CC CTAAAACCCGCCACATCT-3′, ND1-R: 5′-GCGATGGTGAGAGCTAAGGT-3′; ND4-F: 5′-CCATTCTCCTCCTATCCCTCAAC-3′, ND4-R: 5′-CCATTCTCCTCCTATCCCTCAC-3′; cytochrome b-F: 5′-CACGATTCTTTACCTTTCACTTCA TC-3′, and cytochrome b-R: 5′-TGATCCCGTTTCGTGCAAG-3′.

### Electrophysiology

Whole-cell recordings were obtained from neurons with patch pipettes pulled from borosilicate glass (outer diameter, 1.5 mm; inner diameter, 0.86 mm) pulled on a horizontal puller (P-97, Sutter Instruments) and filled with intracellular solution that contained, in mM: k-gluconate, 130; KCl, 6.3; EGTA, 0.5; MgCl_2_, 1.0; HEPES, 10; Mg-ATP, 4.0; Na-GTP, 0.3. pH was adjusted to 7.30 with KOH; osmolality was adjusted to 285 mOsm with 30% sucrose. When filled with internal solution, pipettes had a resistance of 5–7 MΩ. Calculated chloride equilibrium potential was −73 mV. Unless otherwise specified, chemicals were purchased from Sigma-Aldrich (St. Louis, MO, USA).

Recordings were performed with a MultiClamp 700B amplifier (Molecular Devices, Sunnyvale, CA) using pCLAMP 10 software. Pipette capacitance and series resistance compensation (bridge balance) were applied throughout current-clamp experiments, with minor bridge balance re-adjustments allowed as required. Signals were low-pass filtered at 10 kHz and sampled at 20–50 kHz and digitized using a Digi data 1550A 16-bit D/A converter (Molecular Devices). Reported values for membrane potential (*V*_m_) and action potential threshold are not corrected for the liquid junction potential.

Spontaneous resting membrane potential (*V*_m_) was determined 2 min after break-in as the average membrane potential during a 1-s sweep with no current injection. For cells that were spontaneously active, this was measured in the inter-spike interval. Input resistance (*R*_m_) was calculated as the slope of the linear fit to the plot of the *V*–*I* relation derived from small subthreshold current steps at/around resting membrane potential. Action potential threshold was calculated as the voltage at which the first derivative (d*V*/dt) of the AP waveform reached 10 mV/ms. Action potential half-width (AP ½-width) is defined as the width of the AP (in ms) at half-maximal amplitude, calculated using AP threshold and the peak of the AP. Action potential after-hyperpolarization (AHP) amplitude is calculated as the depth of the after discharge potential (in mV) relative to AP threshold.

Maximal steady-state firing frequency is the maximal mean firing frequency in response to a current injection at which there are no AP failures, with spikes defined as having a clear AP threshold as per above, amplitude of 40 mV or higher, and overshooting −10 mV. Maximal instantaneous firing frequency is the inverse of the smallest inter-spike interval at maximal current step injection.

Cutoff frequency (Hz) is defined as the frequency after which a cell can no longer produce action potentials (i.e., there are action potential failures) in response to repetitive stimulation for 10 s.

Experimental design: Data from 7–10 cells per line were included in the analysis.

### Statistical analysis

Experimental “*N*” refers to the number of iPSCs lines from different individuals of each group (control = 5; 22qSZ = 4) used in each experiment.

Data were presented as mean ± standard error of the mean (SEM). Unpaired two-tailed Student’s *t*-test and Chi-square were used for data analysis. *p* < 0.05 was considered a significant difference. Data were analyzed and graphed with GraphPad Prism version 8.

## Results

### Human iPSC-derived neurons (iNrns) from subjects with 22q11DS and SZ have reduced ATP levels and ETC activity

**Fig. 1:**
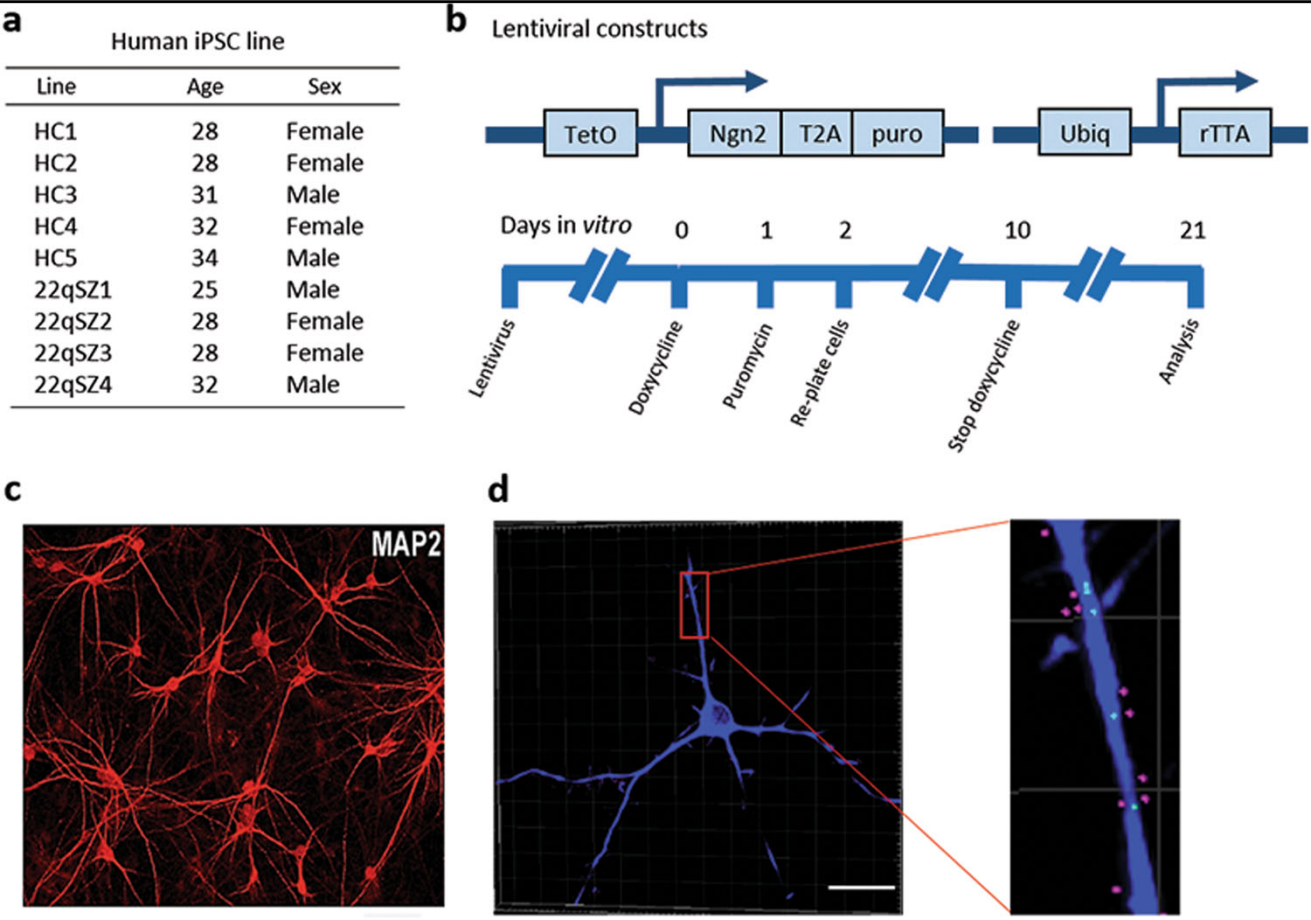
iPSC lines and induced neuronal differentiation protocol used in this study. **a** Table showing subject characteristics. **b** Lentiviral constructs and timeline used to induce neuronal differentiation. Doxycycline is discontinued at 10 days in vitro (DIV). **c** Epifluorescence image of a DIV21 culture, from a 22qSZ line, with fluorescence immunodetection of the neuronal dendritic marker MAP2. **d** Confocal image processed by deconvolution and Imaris software showing apposition of MAP2 (blue) with the excitatory presynaptic protein vGLUT (green), and the excitatory postsynaptic protein PSD95 (red). Scale bars: 50 µm in **c**, 30 µm in **d**.

To investigate the hypothesis that the pathobiology of SZ in the context of 22q11DS could include mitochondrial dysfunction in neurons, we studied five iPSC lines from healthy controls and four lines from subjects with 22q11DS and SZ (22qSZ) that have been published previously^17^ (Fig. 1a). These lines were differentiated via the induced expression of neurogenin 2 (Fig. 1b), a well-established protocol that rapidly and reproducibly generates a relatively homogenous population of excitatory projection neuron-like cells (Fig. 1c, d and see also Fig. S1)^19^.

**Fig. 2:**
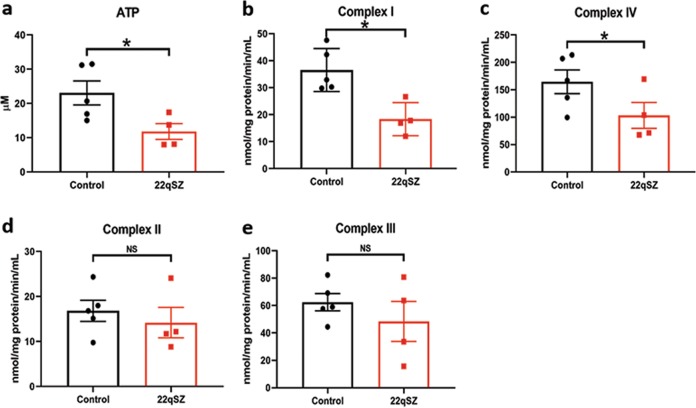
iNrns from subjects with 22qSZ have reduced ATP levels, and reduced ETC complexes I and IV activity. **a** ATP level measured by luminescence detection assay at differentiation day 21. Relative to controls, the 22q11DS group had a nearly 50% reduction of ATP level. **b–e** Mitochondrial complex I (NADH–ubiquinone oxidoreductase) (**b**); complex IV (cytochrome c oxidase) (**c**); complex II (succinate–ubiquinone oxidoreductase) (**d**); and complex III (ubiquinone–cytochrome c oxidoreductase) (**e**) were measured in day21 iPSC-derived neurons. Relative to controls, the 22qSZ group had significant reduction of complexes I and IV enzyme activity, but not of complex II or III. *N* = 5 for control, *N* = 4 for 22qSZ; * indicates *p* < 0.05, NS—not significant.

We first examined ATP production, finding a roughly 50% reduction in the 22qSZ neurons (Fig. 2a). To explore potential causes of this decrease, we next investigated enzymatic activity in the ETC. Both complexes I and IV exhibited significantly reduced activity in 22qSZ neurons (Fig. 2b, c); however, complexes II, III, and V (ATP synthase) enzyme activity was unchanged (Fig. 2d, e and Fig. S2).

While all 13 of the mitochondrial DNA-encoded proteins function in the ETC, they are not evenly distributed^23^. The two ETC complexes with significant reductions of activity in the 22qSZ group, complexes I and IV, have seven and three mitochondrial-encoded proteins, respectively. In contrast, complexes II, III, and V have zero, one, and two of these proteins, respectively. The correlation of complex activity reductions to their number of mitochondrial-encoded proteins raises the possibility that an abnormality in mitochondrial translation could be influencing these phenotypes^24^. That possibility was already implicated by the fact that one of the genes deleted in the 22q11DS, *MPRL40*, encodes for a subunit of the mitochondrial ribosome^25^.

### iNrns from subjects with 22qSZ have reduced levels of mitochondrial-encoded proteins

**Fig. 3:**
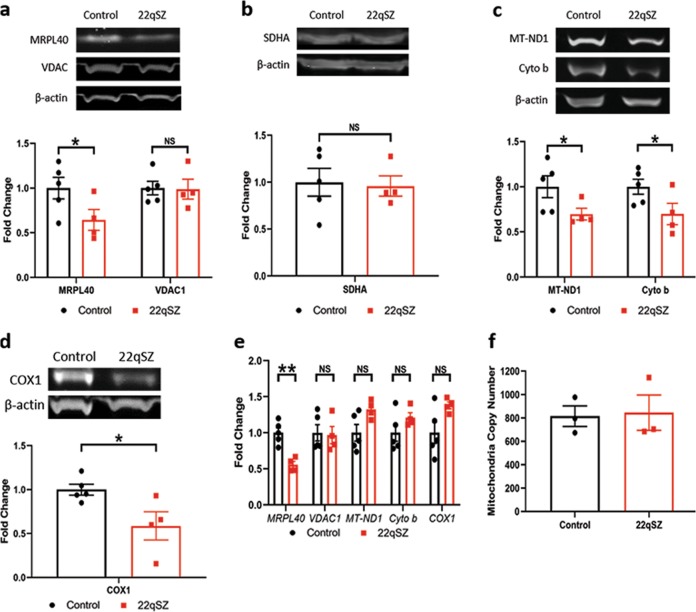
Reductions in mitochondrial-DNA encoded protein in 22qSZ. **a–d** Protein levels, by western blot and relative to β-actin, of MRPL40, VDAC, SDHA, MT-ND1, cytochrome b, and COX1 in iPSC-derived neurons. While nuclear-encoded VDAC and SDHA were unchanged, the mitochondria-encoded proteins MT-ND1, cytochrome b, and COX1 were significantly reduced. **e** Quantitative PCR of MRPL40, VDAC, MT-ND1, cytochrome b, and COX1 shows decreased expression of MRPL40 but no change of VDAC, MT-ND1, cytochrome b, and COX1 in 22qSZ relative to control. **f** No difference of mitochondrial DNA copy number between 22qSZ and control in iPSC-derived neurons at differentiation day 21. *N* = 5 for control, *N* = 4 for 22qSZ; * indicates *p* < 0.05. NS—not significant.

Since MRPL40 (mitochondrial ribosomal protein L40) assists in protein translation of mitochondria encoded genes, we examined mRNA and protein levels encoded by nuclear and mitochondrial genes that generate mitochondrial proteins. As expected, MRPL40 was reduced at the protein (Fig. 3a) and at the mRNA (Fig. 3e) levels^17^. Importantly, neither protein (Fig. 3a) nor mRNA (Fig. 3e) levels of the nuclear encoded voltage-dependent anion channel (VDAC) were altered in neurons from the 22qSZ group. The nuclear encoded complex II protein SDHA was also unaltered (Fig. 3b), as was the mtDNA copy number (Fig. 3f). Together, these results suggest that there are no gross alterations in mitochondrial mass or DNA in 22qSZ group relative to controls.

Remarkably, mitochondrial DNA-encoded protein levels, including those for MT-ND1 (complex I), cytochrome b (complex III), and COX1 (complex IV) were significantly reduced in neurons from the 22qSZ lines (Fig. 3c, d). However, the mRNAs for these proteins were unaltered (Fig. 3e). Taken together, these results suggest that the reduction of ATP in neurons from the 22qSZ-derived lines is secondary to reduced levels of mitochondrial-encoded proteins.

### Heterozygosity for a truncating mutation in *MRPL40* reduces mtDNA-encoded protein expression and neuronal ATP

**Fig. 4:**
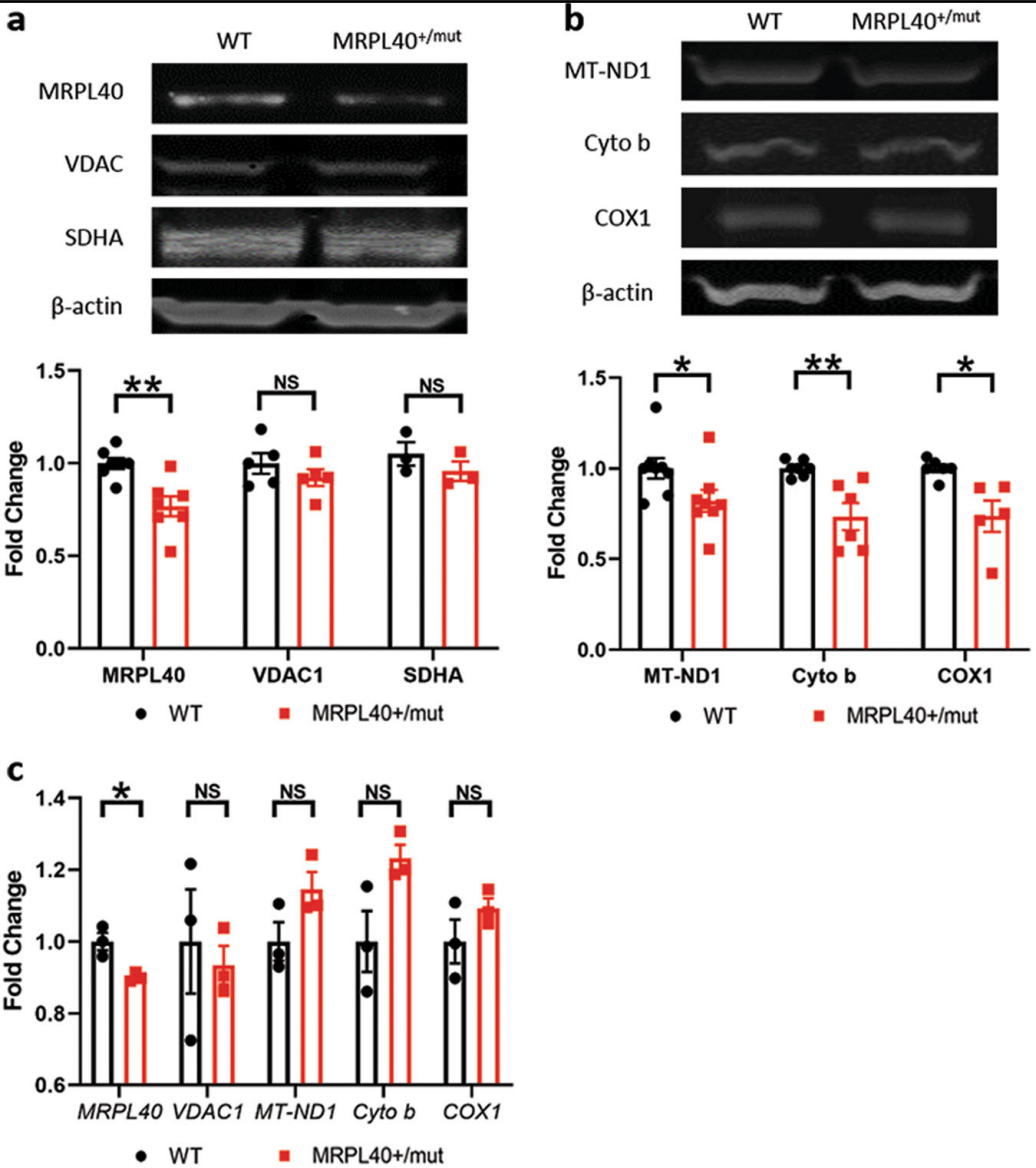
Reduction in mitochondrial-DNA encoded proteins in a *MRPL40* heterozygous line. **a**, **b** Western blot of MRPL40, VDAC, SDHA, MT-ND1, cytochrome b, and COX1 from iPSC-derived neurons at differentiation day (DD) 21. In the *MRPL40*^+/mut^, there is decreased expression of MRPL40, and the mitochondrial DNA-encoded proteins MT-ND1, cytochrome b, and COX1, but no differences in the nuclear-encoded proteins VDAC and SDHA. **c** Quantitative PCR of *MRPL40*, *VDAC*, *MT-ND1*, *Cytochrome b* and *COX1*. In *MRPL40*^+/mut^, there is a small but significant decrease in expression of *MRPL40*, and no change in levels of the other transcripts. * = *p* < 0.05, NS—not significant. For **a** and **b**, MRPL40 (*N* = 7), VDAC1 (*N* = 5), SDHA (*N* = 3), MT-ND1 (*N* = 8), cytochrome b (*N* = 6), and COX1 (*N* = 5). For **c**, *N* = 3.

To test whether haploinsufficiency for *MRPL40* alone is sufficient to compromise ATP levels and mitochondrial protein translation in human neurons, a loss of function mutation was introduced into one allele of *MRPL40* in an iPSC line from a healthy control (Fig. S3). iNrns generated from this line have decreased MRPL40 protein (Fig. 4a) as well as mRNA (Fig. 4c). As found above with the 22qSZ iPSC-derived neurons compared to the healthy control group (Fig. 3), neurons from the *MRPL40*^+/mut^ line showed no change in the nuclear encoded mitochondrial proteins VDAC or SDHA relative to its isogenic control (Fig. 4a). However, MT-ND1, cytochrome b, and COX1 were again significantly reduced at the protein level (Fig. 4b), but not at the RNA level (Fig. 4c).

**Fig. 5:**
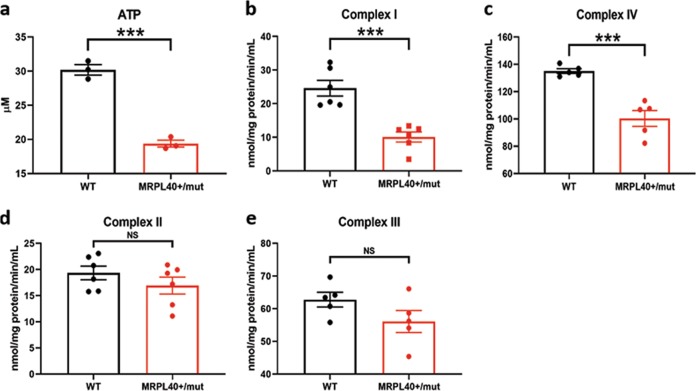
Neurons derived from *MRPL40*^+/mut^ cell show decreased OXPHOS complex I and IV activity and reduced ATP level. **a** Bar graph shows reduced ATP level in the *MRPL40*^+/mut^ neurons (*N* = 3). **b–e** In *MRPL40*^+/mut^ neurons, there is also reduced activity in mitochondrial complex I (**b**, *N* = 6) and complex IV (**c**, *N* = 5), while complex II (**d**, *N* = 6) and complex III (**e**, **N** = 5) activity was not affected. * Indicates *p* < 0.05, NS—not significant.

These results suggest that reduction of MRPL40 results in reduced translation of mitochondrial proteins. To determine whether the haploinsufficiency mutation for *MRPL40* is also sufficient to alter mitochondrial function, ATP levels and ETC complex activities were assessed. Similar to findings from the 22qSZ iNrns, there was a roughly 30% decrease in ATP in iNrns from the *MRPL40*^+/mut^ line (Fig. 5a). Complex I and IV activities were also significantly reduced (Fig. 5b, c), but not complexes II and III (Fig. 5d, e). Taken together, these results suggest that *MRPL40* haploinsufficiency is likely to be an important contributor to the mitochondrial phenotypes identified in iNrns from the 22qSZ group and that the reduction of ATP in iNrns from the 22qSZ-derived lines is secondary to reduced mitochondrial protein translation.

### Neurons from subjects with 22qSZ show different cutoff frequency and similar synapse density as controls

The presence of reduced ATP levels in neurons from 22qSZ patients raises the question of whether these neurons have functional deficits. To test this possibility, cells were plated onto glass coverslips. Rat astrocytes, which greatly increase synaptogenesis in these cultures, were added at day 5. The media was changed to Brainphys at day 21, and then whole cell patch-clamp recordings were performed after 5 weeks in vitro (Fig. S1a). Multiple parameters that could be indicative of neuronal maturational state were unchanged between the 22qSZ and controls, including resting membrane potential (Rm), input resistance, action potential half-width and max steady-state firing frequency (Table S1). On the other hand, measures of parameters thought to test bioenergetics, such as cutoff frequency, show significant difference in 22qSZ versus controls. In addition, synapse density, measured by confocal microscopy of immunofluorescent-labeled synapses along MAP2-labeled dendrites, was not significantly different between the groups (Fig. S1c). In sum, while the 22qSZ neurons have less tendency to respond to break through of the patch-pipette with a spike train, by multiple intrinsic electrophysiological measures as well as synapse density analysis, the 22qSZ iNrns had well-established neuronal characteristics and were not distinguishable from controls at about day 60.

## Discussion

SZ primarily evolves from the interacting influences of multiple risk-alleles whose effects on brain development can also be influenced by both the prenatal and postnatal environment^26,27^. One of the strongest genetic risk factors for SZ is the hemizygous microdeletion at chromosome 22q11.2 that imparts a 25% risk, or about 25 times that of the general population^28^. Since nine of the roughly 45 genes deleted in 22q11DS generate proteins affect mitochondrial function^11,12^, we examined this function in iPSC-derived neurons. We found that ATP levels were reduced in iPSC-derived neurons from patients with 22qSZ, a phenotype that appears to be caused primarily by reduced activity of complexes I and IV. Interestingly, neurons from the 22qSZ lines also had reduced levels of several mitochondrial-encoded proteins, but not of nuclear encoded proteins. Since complexes I and IV have the highest number of mitochondrial-encoded proteins, these findings raised the possibility that the 22q11DS is associated with reduced neuronal protein synthesis, an idea supported by the presence of *MRPL40* in the deleted region. We thus edited a control iPSC line to be hemizygous for *MRPL40*. Relative to its isogenic control, and like the 22qSZ lines relative to their controls, the *MRPL40* hemizygous neurons have normal levels of mitochondria proteins encoded by nuclear genes, but decreased levels of proteins encoded by the mitochondrial DNA, decreases in ETC complexes I and IV activities, and decreased ATP level. We conclude that hemizygosity at 22q11.2 is associated with decreased neuronal ATP levels, and that this phenotype is most likely related to decreased mitochondrial protein synthesis. Unfortunately, our attempts to quantify mitochondrial protein synthesis in mature iNrns have been unsuccessful. We believe this is the first demonstration of a SZ-related mutation resulting in a mitochondrial deficit in human neurons.

A key aspect of any study involving the use of human iPSCs to study disease is the cell type chosen. Here, human iPSC lines were differentiated via the induced expression of neurogenin 2, which rapidly generates a relatively homogenous population of excitatory projection neuron-like cells (iNrns)^19^. An advantage of this approach is that reasonably mature neurons can be generated in a matter of weeks, with high degree of consistency across lines. A disadvantage of this approach for the current study is that neurogenin 2 expression is induced by doxycycline, an inhibitor of bacterial mitochondrial protein synthesis that can also affect mammalian mitochondria^29^. Of note, mitochondrial assays were run 11 days following stoppage of the doxycycline in the neuronal induction protocol, but the possibility remains that doxycycline, or even the puromycin exposure for 1 day at the start of the protocol, could be unmasking a vulnerability in the 22qDS and the *MRPL40* hemizygous iNrns.

Since mitochondrial gene and protein expression, as well as functional properties, are likely to differ across cell types, the relative uniformity provided by the Ngn2 protocol is another major advantage. In fact, while the lack of neuronal phenotypes such as input synaptogenesis and electrophysiological measures is disappointing, since such phenotypes could secondarily influence mitochondrial function, the high similarities in properties between iNrns from the 22qSZ and control groups strengthens our confidence that the mitochondria phenotypes themselves are not epiphenomena. That we replicated these phenotypes in the hemizygous *MRPL40* line relative to its isogenic control, further bolsters this contention. In future studies, it will be important to assess mitochondrial phenotypes in longer duration culture systems, transplantations, and well as with electrophysiological conditions such as limited ATP and EGTA in the recording solution that might unmask relevant phenotypes. It would also be interesting to study haploinsufficiency for *MRPL40* affects calcium signaling, as studied in mouse hippocampal neurons^16^. In addition, other cell types that can show SZ-related phenotypes should be tested, including astrocytes, microglia, and cINs. The “fast-spiking” cIN has more mitochondria in its pre-synaptic terminals than other cortical neurons^30^, and has otherwise been associated with high energy requirements, susceptibility to oxidative stress, and SZ^31,32^. However, to date the fast-spiking subclass of cINs has not been definitively generated, in a highly enriched preparation, from human stem cells.

So what bearing might these findings have to understanding SZ? Of course, we cannot conclude that our findings relate to the SZ-related symptoms of the 22q11.2 subjects in our study. Important results would be obtained from future studies involving the comparison of iPSCs from 22q11DS subjects with versus without SZ. It also bears mention that *MRPL40* haploinsufficiency alone is unlikely to account for all mitochondrial deficits associated with 22qDS or with SZ^5,13,14,33^, but multiple lines of evidence suggest that these findings are indeed important. First, transgenic mice hemizygous for *MRPL40* show deficits in working memory, a SZ-related phenotype, as well as alterations in hippocampal short-term potentiation^16^. Second, alterations in the expression of mitochondrial-functioning genes have been identified in a human post-mortem, laser-capture microdissection study of cortical pyramidal neurons^5^. Interestingly, these include downregulation of *MRPL36*, *MRPL48*, and *MRPS6*, raising the possibility that a mitochondrial translation deficit could be an important aspect of SZ outside of the 22q11DS context^5^. In addition, *MRPL18* was highlighted as a gene associated with psychosis based on whole-blood RNA expression in 22q11DS subjects^34^. Third, the capacity that we have demonstrated to study mitochondria defects in iPSC-derived neurons suggests that this system will be amendable to increasingly refined and mechanistic studies on interactions between mitochondria genetics and neuropsychiatric risk, and resilience.

## Supplementary information

Supplemental figure 1–4, table 1 and legends
