# Supplementary material for: Mitochondrial deficits in human iPSC-derived neurons from patients with 22q11.2 deletion syndrome and schizophrenia
Source: Transl Psychiatry. 2019 Nov 18;9:302. doi: 10.1038/s41398-019-0643-y (PMC6861238; doi:10.1038/s41398-019-0643-y)
Supplement: Supplementary file 1 — Supplemental figure 1–4, table 1 and legends [file 41398_2019_643_MOESM1_ESM.pdf]

Figure S1

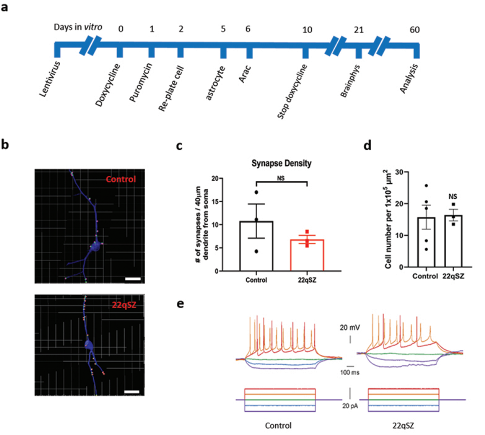
a
Days in vitro
0
1
2
5
6
10
21
60
Lentivirus
Doxycycline
Puromycin
Re-plate cell
astrocyte
Arac
Stop doxycycline
Brainphys
Analysis
b
Control
22qSZ
c
Synapse Density
NS
# of synapses / 40μm dendrite from soma
Control
22qSZ
d
Cell number per 1x10^5 μm^2
NS
Control
22qSZ
e
20 mV
100 ms
20 pA
Control
22qSZ

Figure S2

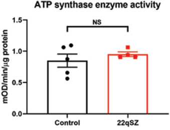
ATP synthase enzyme activity
NS
mOD/min/μg protein
1.5
1.0
0.5
0.0
Control
22qSZ

Figure S3

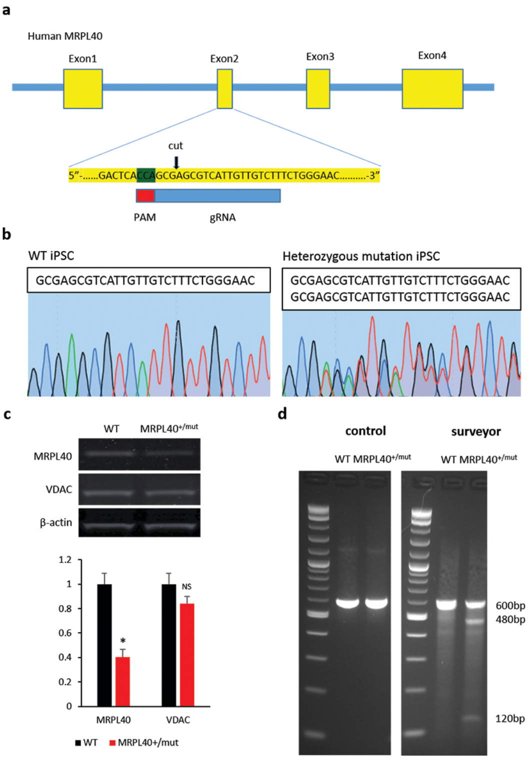
a
Human MRPL40
Exon1
Exon2
Exon3
Exon4
cut
5"-......GACTCACCAGCGAGCGTCATTGTTGTCTTTCTGGGAAC..........-3"
PAM
gRNA
b
WT iPSC
GCGAGCGTCATTGTTGTCTTTCTGGGAAC
Heterozygous mutation iPSC
GCGAGCGTCATTGTTGTCTTTCTGGGAAC
GCGAGCGTCATTGTTGTCTTTCTGGGAAC
c
WT
MRPL40+/mut
MRPL40
VDAC
β-actin
1.2
1
0.8
0.6
0.4
0.2
0
NS
*
MRPL40
VDAC
WT
MRPL40+/mut
d
control
surveyor
WT MRPL40+/mut
WT MRPL40+/mut
600bp
480bp
120bp

Figure S4

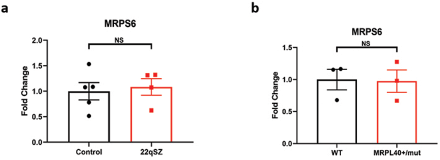
a
MRPS6
NS
Fold Change
2.0
1.5
1.0
0.5
0.0
Control
22qSZ
b
MRPS6
NS
Fold Change
1.5
1.0
0.5
0.0
WT
MRPL40+/mut

Table S1

Electrophysiological properties of control VS. 22qSZ

	Intrinsic properties		Single APs	Repetitive firing	
	V_m	*Rm*	½-width	Max Steady-State Firing frequency	Cutoff frequency
	mV	Mohms	ms	Hz	Hz
Control	-53.1 ± 2.3	429 ± 10	2.8 ± 0.4	10.9 ± 1.7	28.1 ± 1.4
22qSZ	-51.5 ± 3.2	539 ± 77	2.2 ± 0.2	15 ± 4.1	38.1 ± 2.3
P value	0.69	0.16	0.29	0.35	0.011

Supplemental figure legends

Figure S1. Synaptic density in iNrns from 22q11DS with schizophrenia. **a** schema of the experimental design for the synapse density and whole cell recording at day 60. **b** Confocal image processed by deconvolution and Imaris software showing apposition of MAP2 (Blue) with the excitatory presynaptic protein vGLUT (Green), and the excitatory postsynaptic protein PSD95 (Red) in control and 22qSZ. Scale bar: 20 µm. **c** Bar graph shows no significant alternation of synapse density between control and 22qSZ group. (N=3). **d** Bar graph shows no difference of neuron density between cultures of control and 22qSZ group in which the synapse density and whole-cell recordings were measured. (N=3-5). **e** Tracing show representative responses of neurons to 20pA current steps.

Figure S2. Bar graph shows no significant change of ATP synthase activity in iPSC-derived neurons from 22qSZ (N=4) comparing with control (N=5).

Figure S3. Generation of an iPSC line haploinsufficient for *MRPL40*. **a** Schematic of CRISPR-Cas9 editing of exon 2. **b** DNA sequence of the mutated line, showing four base pairs in exon 2 deleted on one allele. This 4bp deletion causes a frame shift resulting in a premature stop codon in exon 3. **c** In iPSC cell lysates, western blotting reveals a reduction of MRPL40 but no change in VDAC in the $MRPL40^{+/mut}$ line. No band was detected at the predicted size of the mutated allele, possibly due to nonsense-mediated decay. N=3. **d** SURVEYOR detection assay is an enzyme mismatch cleavage assay, in which DNA is cut at regions of single base mismatches or small insertions or deletions. Corresponding to schematic of CRISPR-Cas9 editing of exon 2, PCR product reveal the expected 600, 480 and 120 bp bands, heterozygous mutation of *MRPL40*.

Figure S4. Real time PCR shows no alteration of *MRPS6* in iPSC-derived neurons from **a** 22qSZ (N=4) verses control (N=5); **b** WT verses $MRPL40^{+/mut}$ line (N=3).

Table S1. Electrophysiological properties between cultured neurons from the control (N=4) and 22qSZ groups (N=3). 7-10 cultured neurons were recorded in each line. Vm-resting membrane potential. Rm-input resistance, action potential (AP) 1/2 width, maximum firing frequency and cutoff frequency.
